# Improved osteogenic differentiation by extremely low electromagnetic field exposure: possible application for bone engineering

**DOI:** 10.1007/s00418-022-02126-9

**Published:** 2022-06-25

**Authors:** Erica Costantini, Guya Diletta Marconi, Luigia Fonticoli, Lisa Aielli, Oriana Trubiani, Thangavelu Soundara Rajan, Jacopo Pizzicannella, Marcella Reale, Francesca Diomede

**Affiliations:** 1 Department of Medicine and Science of Aging, University “G. d’Annunzio”, Via deiVestini, 66100 Chieti, Italy; 2 Department of Medical, Oral and Biotechnological Sciences, University “G. d’Annunzio”, Via dei Vestini, 66100 Chieti, Italy; 3 Department of Innovative Technologies in Medicine and Dentistry, University “G. d’Annunzio”, Via dei Vestini, 66100 Chieti, Italy; 4 Department of Biotechnology, Karpagam Academy of Higher Education, Coimbatore, 641021 India; 5 “S.S. Annunziata Hospital”, ASL 02 Lanciano-Vasto-Chieti, Via dei Vestini, 66100 Chieti, Italy

**Keywords:** Osteogenic differentiation, Bone, Tissue engineering, Low electromagnetic field, Human periodontal ligament stem cells

## Abstract

Human periodontal ligament mesenchymal stem cells (hPDLSCs) are a promising cell type model for regenerative medicine applications due to their anti-inflammatory, immunomodulatory and non-tumorigenic potentials. Extremely low-frequency electromagnetic fields (ELF-EMF) are reported to affect biological properties such as cell proliferation and differentiation and modulate gene expression profile. In this study, we investigated the effects of an intermittent ELF-EMF exposure (6 h/day) for the standard differentiation period (28 days) and for 10 days in hPDLSCs in the presence or not of osteogenic differentiation medium (OM). We evaluated cell proliferation, de novo calcium deposition and osteogenic differentiation marker expression in sham and ELF-EMF-exposed cells. After ELF-EMF exposure, compared with sham-exposed, an increase in cell proliferation rate (*p* < 0.001) and de novo calcium deposition (*p* < 0.001) was observed after 10 days of exposure. Real-time PCR and Western blot results showed that COL1A1 and RUNX-2 gene expression and COL1A1, RUNX-2 and OPN protein expression were upregulated respectively in the cells exposed to ELF-EMF exposure along with or without OM for 10 days. Altogether, these results suggested that the promotion of osteogenic differentiation is more efficient in ELF-EMF-exposed hPDLSCs. Moreover, our analyses indicated that there is an early induction of hPDLSC differentiation after ELF-EMF application.

## Introduction

Mesenchymal stem cells (MSCs) are stromal cells characterized by the ability to undergo self-renewal, guaranteeing a pool of stem cells, and to differentiate into mesodermal, ectodermal and endodermal cell lineages. The latter enables them to subsequently differentiate into osteoblasts, adipocytes, chondrocytes (mesodermal lineage), neurons (ectodermal lineage) and liver cells (endodermal lineage) under specific in vitro conditions (Pizzicannella et al. 2021b). MSCs express cluster of differentiation (CD) surface markers that allow them to be characterized and distinguish them from the hematopoietic cells, especially by evaluating the lack of the expression of CD14, CD34, CD45 and human leukocyte antigen (HLA-DR) and positivity for CD73, CD90, nestin and βIII tubulin (Tuj-1) as neuronal markers, CD146 and CD105 asendothelial markers, transforming growth factor beta (TGF β) receptor and several integrins (Soundara Rajan et al. 2017). These adult stem cells can be isolated from different tissues. Among these the most used is the bone marrow, which represents the gold standard especially because of the ease of collection. In addition, other sources are adipose tissue, peripheral blood and neonatal tissues (umbilical cord, placenta, amniotic fluid and amniotic membrane) (Ullah et al. 2015). It was discovered that MSCs can be isolated from menstrual blood and endometrium (Hida et al. 2008; Musina et al. 2008; Toyoda et al. 2007). MSCs appear to exhibit different characteristics depending on their source and the microenvironment from which they are isolated (Elahi et al. 2016). Several research teams have tried to optimize the clinical application of MSCs isolated from the most common sources mentioned above. For example, Talwadekar et al. found that the properties of placental MSCs (P-MSCs) were superior to cord-derived MSCs (C-MSCs), while Nagaishi et al. have shown that umbilical cord MSCs have a better therapeutic effect than bone marrow-derived MSCs for certain diseases such as diabetes (Talwadekar et al. 2015; Nagaishi et al. 2017). Therefore, the source of MSC isolation is also important. In this regard, it is particularly interesting that in recent years new alternative tissue sources have been identified from which adult MSCs can be obtained, such as the oral cavity (Andrukhov et al. 2019). The oral cavity is one of the major sources of MSCs due to its easy accessibility. In particular, from the oral cavity different MSC populations can be isolated: stem cells from human pulp (hDPSCs), stem cells from human exfoliated deciduous teeth (SHEDs), stem cells from human periodontal ligaments (hPDLSCs), stem cells from human apical papilla (hAPSCs), human dental follicle stem cells (hDFSCs) and human gingival mesenchymal stem cells (hGMSCs) (Trubiani et al. 2012). The periodontal ligament, a connective tissue present between the inner wall of the alveolar bone socket and the cement, provides structural support of the tooth and promotes the nutrition, repair and the maintenance of tissue homeostasis (Seo et al. 2004b). It is widely reported that within the periodontal ligament tissue other than adult MSCs, cells such as osteoblasts, fibroblasts, and epithelial and endothelial cells can be isolated. The hPDLSCs show the typical characteristics of MSCs obtained from different tissues. Indeed, these cells can divide countless times without losing their phenotype and their karyotype, can differentiate toward different cell lines such as osteocytes adipocytes and chondrocytes, can express mesenchymal surface markers and are negative for hematopoietic markers. In addition, hPDLSCs exhibit anti-inflammatory, immunomodulatory and non-tumorigenic abilities (Marconi et al. 2021). All these features, together with their easy accessibility, make hPDLSCs one of the most studied MSC populations. To date, these cells represent an excellent model for studies in the field of regenerative medicine and dentistry. Moreover, it is widely reported that these cells play an important role in bone regeneration processes. For example, hPDLSCs improved the osseointegration process by promoting the formation of an interface between the bone and devices used for dental implants (Diomede et al. 2020). Therefore, hPDLSCs are widely used as a model for the development of new therapies in bone trauma or pathologies such osteoporosis, arthritis, osteonecrosis and periodontitis (Diomede et al. 2017).

Orthopedic and dental bone defects result from trauma, infections, neoplasms, congenital conditions or the normal aging processes. For the resolution of these alterations and the recovery of functions, it is necessary to use strategies to ensure an ideal structural environment for the cells participating in the bone healing process. A massive use of MSCs has been considered in tissue engineering and cell therapy (Rosenbaum et al. 2008) because of the pluripotent cell characteristics, such as the ability to differentiate into osteogenic, adipogenic and chondrogenic cells upon in vitro exposure to different inducers (Seo et al. 2004a; Xu et al. 2009). Due to the high-cost, highly sensitive and easily degradable systems of specific differentiating agents, such as TGF-β for bone cells (Elsafadi et al. 2018) or prostaglandin (PG)E2 for vascular cells differentiation (Zhu et al. 2011), it is important to find cheaper and stable systems to induce stem cell differentiation to broaden the already promising applications.

Studies on the effects of electromagnetic field (EMF) exposure in human health have shown the bioelectromagnetic interactions between non-ionizing radiation and biological systems evaluated in in vivo and in vitro studies. Today, EMF treatments are widely used in neurology, psychiatry, rheumatology, dermatology and bone formation, which can also be obtained after biophysical stimulation such as mechanical stresses, ultrasounds and electric fields. Thus, EMF can represent new strategies for maintaining health (D’Angelo et al. 2015).

EMF applications and effectiveness are variable in relation to the time of exposure, waveform, frequency, amplitude, cell type and cellular response modulation in proliferation, differentiation, cytokine and growth factor expression, and nitric oxide signaling (Gualdi et al. 2021). In particular, pulsed electromagnetic fields (PEMF) are reported to have considerable application as a safe biophysical additional treatment for bone fractures and tissue repair and only recently in relation to stem cells differentiation. Data from the literature demonstrated the ability of PEMF stimulation to enhance osteogenic differentiation of MSCs, inducing the early expression of osteogenic genes and protein expressions (Wang et al. 2017a; Varani et al. 2021), suggesting a possible application in bone repair. Moreover, exposure to extremely low frequency (ELF)-EMFs from power lines and commonly used devices has been reported to have a possible effect on stem cell differentiation toward neurogenic (Ma et al. 2014; Kim et al. 2013) and osteogenic phenotypes (Liu et al. 2013). However, the underlying cellular mechanisms on ELF-EMFs-mediated MSCs differentiation remain poorly understood. Accordingly, knowledge about the underlying mechanisms will help in the development of innovative and advanced therapies aimed to reconstruct irremediably damaged tissues and organs through the clinical application of adult MSCs.

In accord with one of the main objectives of current research to stop the progression of different pathologies and to induce the regeneration of injured tissues by broadening the frontiers of regenerative medicine, the aim of the present study was to determine whether intermittent exposure to 50-Hz ELF-EMF for 6 h/day affected proliferation and differentiation of hPDLSCs.

## Materials and methods

### Exposure system

**Fig. 1:**
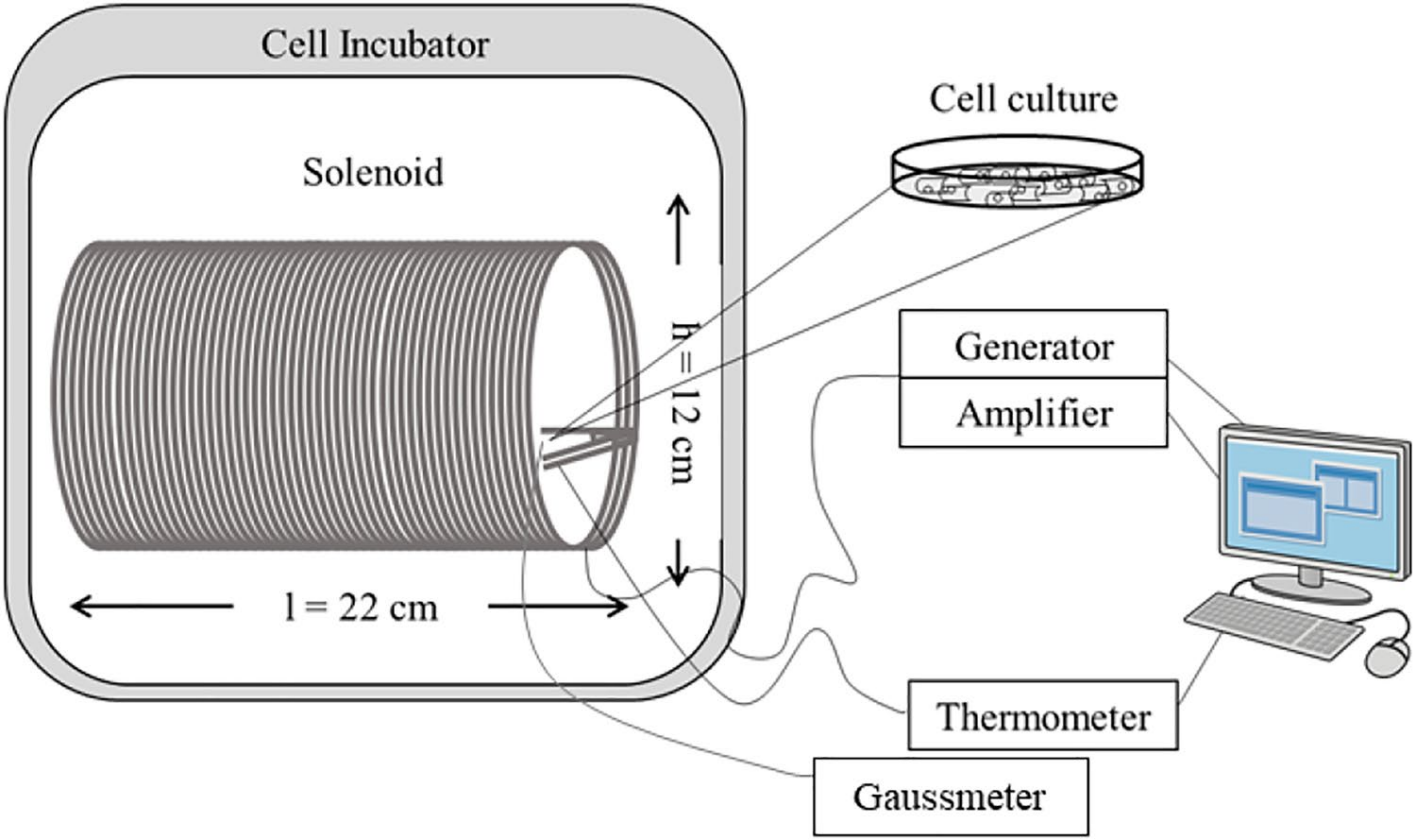
ELF-EMF exposure system. The exposure system is composed of the power supply, power amplifier and coil solenoid. To verify the field stability and temperature, a gaussmeter and thermometer were respectively used. All the components are computer controlled. The sinusoidal field generated is of 1 mT rms 50 Hz and 42 mV

The system used for the experiments consisted of two identical exposure chambers, one for active field and one for sham exposure. The active ELF-EMF contained 1mT rms of a sinusoidal 50-Hz field produced by an electromagnetic generator (Agilent model, Santa Clara, CA, USA) connected with a power amplifier (model 216; NAD Electronics Ltd, London, UK), an oscilloscope (ISO‐TECH model ISR658; Vicenza, Italy) and a Gaussmeter (MG‐3D; Walker Scientific Inc., Worcester, MA, USA). A current flow of 42 mA passed through a 160-turn solenoid coil (22 cm length, 6 cm radius, copper wire diameter 1.25 × 10 − 5 cm), with a 98% of field homogeneity in the central region. The sham chamber was characterized by the dual-winded coils turned in the opposite direction with identical currents to generate the same thermal load (~ 1 W at 1 mT) but without generation of an electromagnetic field. The magnetic field (MF) lines internally follow the longitudinal path of the solenoid, going in the opposite direction externally and forming a loop. The MF flux density was continuously monitored using a Hall-effect probe connected to the gaussmeter. Both exposure systems were computer controlled and analyzed by SW-U801-WIN software for automated and continuous generation monitoring of coil currents. Temperatures were recorded by a two-channel thermometer (TM-925, Lutron, Coopersburg, PA, USA). No significant temperature changes were observed to be associated with the application of the ELF-EMF field (∆T = 0.1 °C) (Patruno et al. 2020). Figure 1 shows the experimental device used for cell exposure.

Preliminary experiments have been conducted to identify the propagation areas where the cell culture is uniformly covered by the generated ELF-EMF. In the central area of the solenoid, where the field lines are parallel to its length, a field uniformity of 98% was achieved. Therefore, the cell cultures were placed within this region of the solenoid.

The exposure systems were placed in two different commercial CO_2_ incubators (HeraCell 240i, Thermo-Fisher Scientific, Waltham, MA, USA) maintained at 37 ± 0.3 °C in a humidified atmosphere of 5% CO_2_ for the same times and conditions.

### Cell culture establishment

Periodontal ligament tissue biopsies were carried out from five human premolar teeth removed for orthodontic reasons. Selected patients were in a healthy general condition without oral and systemic diseases. Biopsies were collected from the third coronal area of the periodontal ligament using a Gracey curette to remove only the small tissue fragments. Patients enrolled in the present study had signed the informed consent. The present study was approved by the Ethics Committee of G. d’Annunzio University (266/2014). Tissue samples were placed in a petri dish with TheraPEAK™ MSCGM-CD™ Chemically Defined Mesenchymal Stem Cell Medium’s formulation (Lonza, Basel, Switzerland). The culture medium was changed twice a week for 15 days until the hPDLSCs started to migrate spontaneously from tissue fragments. Then, cells were collected by adding 0.1% trypsin-EDTA solution (Lonza, Basel, Switzerland) and subsequently sub-cultured until passages 2 and 3 for the following experiments (Trubiani et al. 2019).

### Cell characterization

The hPDLSC characterization was performed by evaluation of mesenchymal marker expression and their ability to differentiate toward adipogenic and osteogenic lineages. Stem cell markers were identified by flow cytometry. Briefly, the hPDLSCs at passage 2 were detached by 0.1% EDTA trypsin (Lonza, Basel, Switzerland) and resuspended in PBS (Lonza, Basel, Switzerland). Subsequently, the cells were stained with fluorescein isothiocyanate (FITC)-conjugated anti-CD45 and CD105 (Ancell Corp., Stillwater, MN, USA), phycoerythrin (PE)-conjugated anti-CD73, FITC-conjugated anti-CD90 and PE-conjugated anti-CD34 (Beckman Coulter, Fullerton, CA, USA). This labeling led to analyzing the cells using the FACSCalibur flow cytometer (Becton-Dickinson, Mountain View, CA, USA) and CellQuestTM software (Becton-Dickinson). To prevent non-specific fluorescence, isotopes were used as controls. At the end of the analysis, the data obtained were analyzed using the specific software FlowJoTM (Becton-Dickinson) (Diomede et al. 2021).

The osteogenic and adipogenic differentiation ability of hPDLSCs was evaluated by both colorimetric assay and gene expression analysis. Concerning colorimetric detection for osteogenic differentiation, the cells were cultured for 28 days with hMSC Osteogenic Differentiation Medium (Lonza, Basel, Switzerland). At the end of 21 days, osteogenic differentiation was assessed by Alizarin Red S solution staining (Sigma-Aldrich, Milan, Italy) able to identify calcium deposits. The adipogenic differentiation was carried out by culturing the hPDLSCs for 28 days with hMSC Adipogenic Differentiation Medium (Lonza, Basel, Switzerland). Subsequently, Oil Red O solution (Sigma-Aldrich, Milan, Italy) was used to show the presence of lipid droplets at the cytoplasmic level. The completed osteogenic and adipogenic differentiation was highlighted by Leica DMIL inverted light microscopy (Leica Microsystem, Milan, Italy). Subsequently, gene expression analysis was performed through RT-PCR set-up using TaqMan Gene Expression Assays and Taq-Man Universal PCR Master Mix (Applied Biosystems, Foster City, CA, USA). This analysis allowed to identify some osteogenic and adipogenic markers, confirming that the hPDLSCs were correctly differentiated. Specifically, Runt-related transcription factor-2 (RUNX-2 Hs00231692_m1) and alkaline phosphatase (ALP Hs01029144_m1) were evaluated for osteogenic differentiation, while fatty acid-binding protein 4 (FABP4 Hs01086177_m1) and peroxisomal proliferator-activated *γ* receptor (PPARγ Hs01115513_m1) were evaluated for adipogenic differentiation. In both cases, beta-2 microglobulin (B2M Hs99999907_m1) (Applied Biosystems) was used to normalize the obtained results (Marconi et al. 2021).

### Experimental design

**Fig. 2:**
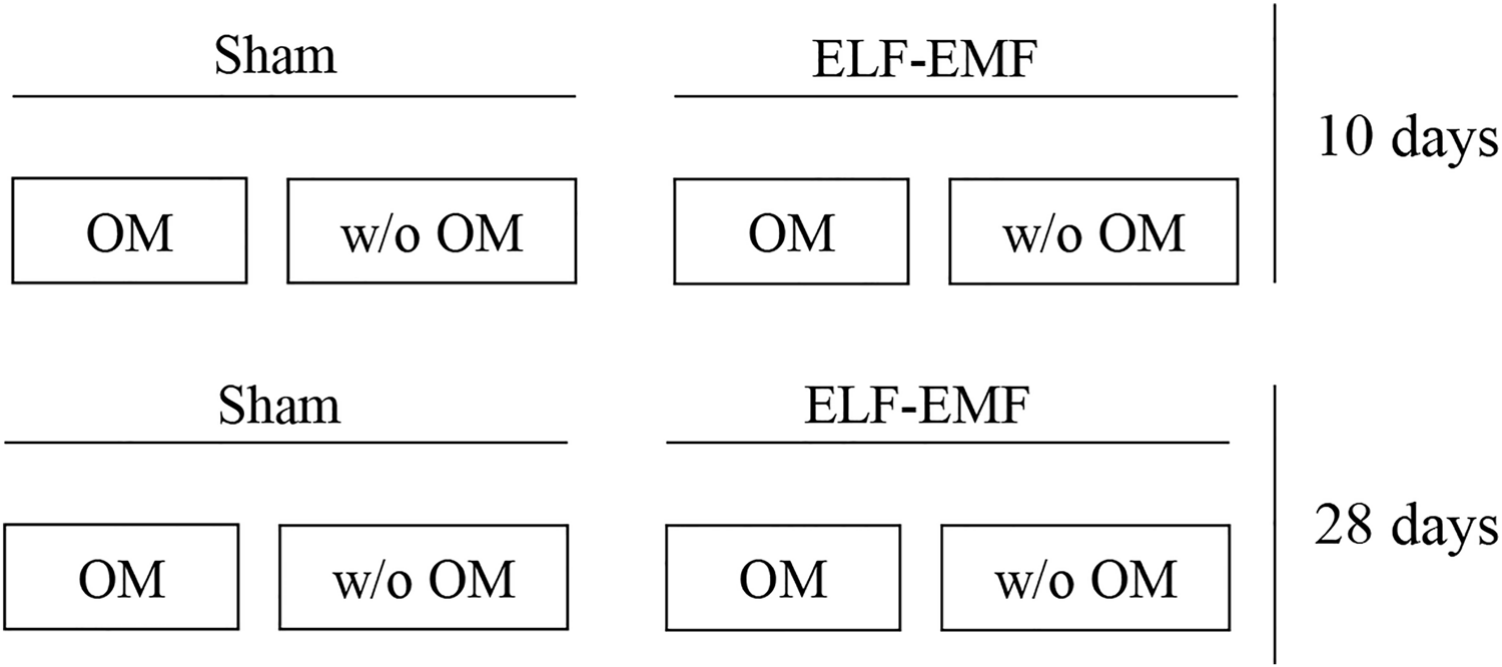
Schematic representation of experimental conditions

All the experiments were performed in duplicate using hPDLSCs at passage 3 (p3). Cells were cultured in the presence of osteogenic medium (OM) or not (without OM) for two different times of treatment, 10 and 28 days. For each treatment, cells were exposed to ELF-EMF or to sham system for 6 h a day (Fig. 2).

### Cell viability and proliferation assay

hPDLSCs were seeded in 96-well flat-bottom plates at a density of 1 × 10^3^ cells/well. After 24 h, cells became confluent, the medium was replaced, and cells were incubated for 6–24 and 48 h in presence of ELF-EMF. Cell viability and proliferation were determined by 3-(4,5-dimethylthiazol-2-yl)-2,5-diphenyltetrazolium bromide (MTT) assay according to the manufacturer's recommendations (Merck KGaA, Darmstadt, Germany). At the end of incubation, MTT reagent was added for 2 h and incubated at 37 °C, and absorbance was assessed at OD590 nm using a Glomax multi-detection reader spectrophotometer (Promega, Milan, Italy). Absorbance data were normalized to the unexposed control group, which was considered 100% of viability.

### Osteogenesis process evaluation in the presence of experimental conditions

The evaluation of calcium deposition and extracellular matrix (ECM) mineralization was obtained by Alizarin Red S staining assay performed at 10 and 28 days of culture in all considered conditions. Samples were washed with PBS, fixed in 10% (v/v) formaldehyde (Sigma-Aldrich) for 30 min and then washed twice with abundant distillated (d) H_2_O prior to the addition of 0.5% Alizarin Red S solution in H_2_O, pH 4.0, for 1 h at room temperature. After incubation under gentle shaking, cells were washed with dH_2_O four times for 5 min. For staining quantification, 800 μl 10% (v/v) acetic acid was added to each well. Cells were incubated for 30 min and were scraped from the plate, transferred into a 1.5-ml vial and vortexed for 30 s. The obtained suspension, overlaid with 500 μl mineral oil (Sigma-Aldrich), was heated to 85 °C for 10 min and then transferred to ice for 5 min, carefully avoiding opening of the tubes until fully cooled, and centrifuged at 20,000×*g* for 15 min. Then, 500 μl supernatant was placed into a new 1.5-ml vial, and 200 μl 10% (v/v) ammonium hydroxide was added (pH 4.1–4.5); 150 μl of the supernatant obtained from the cultures were read in triplicate at 405 nm by a spectrophotometer (Synergy HT, BioTek, Bad Friedrichshall, Germany).

### Immunofluorescence detection

hPDLSCs were cultured in a chamber slide with eight wells (IBIDI, Gräfelfing, Germany) and maintained in all considered conditions. At 10 days, the samples were fixed for 30 min at room temperature with 4% paraformaldehyde in PBS, pH 7.4, and permeabilized with 0.1% Triton-X100 in PBS for 10 min, followed by blocking with 5% skimmed milk in PBS for 30 min. Samples were incubated with primary monoclonal antibody, anti-RUNX-2 (1:200, Santa Cruz Biotechnology, Inc., Dallas, TX, USA), followed by anti-rabbit Alexa Fluor 488 (Molecular Probes, Life Technologies, Monza, MI, Italy). All samples were incubated with Alexa Fluor 568 phalloidin red fluorescence conjugate (1:400) as a marker of the cytoskeleton actin and with TO-PRO staining to highlight cellular nuclei. Samples were observed using a confocal laser scanning microscopy system (Zeiss LSM800, Zeiss, Jena, Germany), equipped with a Plan Neofluar oil-immersion objective (40 ×/1.3 NA). Images were collected using an argon laser beam with excitation lines at 488 nm and a helium-neon source at 543 and 633 nm. Line profile and co-localization analyses were performed offline on images acquired at a resolution of 1024 × 1024 pixel at 12 bit (4096 gray values) using Zen2010 software (Zeiss). The relative fluorescence intensities of RUNX-2 were quantified using NIS-Elements AR imaging software (Nikon). For the counting statistics of immunofluorescence-positive nuclei for ERK and pERK, ten views (100×) were randomly chosen in each experimental group and analyzed using NIS-Elements AR imaging software (Nikon). All the experiments were repeated at least three times. Data are presented as the mean and standard error of the mean (mean ± SEM). The comparison analysis of different groups was done using a one-way analysis of variance followed by a post hoc Bonferroni evaluation using GraphPad Prism5. Differences were termed statistically significant at *p* < 0.05.

### RNA isolation and real-time PCR analysis

hPDLSCs were seeded at 8 × 10^4^ cells/well in six-well plates. Reaching 80% of confluence, culture plates were divided into two groups: (1) OM or (2) no OM for 10 and 28 days. Both plates were exposed or not to ELF-EMF. Cells in each condition were maintained in culture for 28 days, and, after harvesting, total RNA was isolated using the classic phenol–chloroform method. Total RNA was quantified at 260 nm using NanoDrop 2000 ultraviolet-visible (UV–Vis) spectrophotometer (Thermo Fisher Scientific, Waltham, MA, USA); 1 μg of RNA was reverse transcribed to cDNA for 15 min at 42 °C and 3 min at 90 °C to inactivate Quantiscript Reverse Transcriptase, according to the protocol of QuantiTect-Reverse Transcription Kit (Qiagen, Hilden, DE). Real-time PCR was performed in a Mastercycler ep realplex real-time PCR system (Eppendorf, Hamburg, Germany), using PrimeTime Gene Expression Master Mix (IDT, Coralville, IA) to evaluate the gene expression of RUNX-2 and COL1A1, using GAPDH as housekeeping gene. The amplification program consisted of a pre-incubation step for cDNA denaturation (3 min 95 °C), followed by 40 cycles consisting of a denaturation step (0.05 s 95 °C) and an annealing step (0.30 s 60 °C). RT-PCR was performed in two independent experiments, and triplicate determinations were carried out for each sample. Expression levels for each gene were performed according to the 2^−ΔΔCt^ method.

### Western blot analysis

The proteins were collected after 6 h, 24 h and 48 h of culture under sham and ELF-EMF conditions. Moreover, at the end of the osteogenic differentiation, the proteins were isolated from the cellular cultures following the procedure: after 10 and 28 days of culture OM/ELF-EMF, OM/sham, without OM/ ELF-EMF and without OM/sham, the hPDLSCs were lysed with RIPA buffer (Thermo Fisher Scientific) and collected by scraping. At this point, the lysate was centrifugated at 16,000×*g* × 15 min at 4 °C allowing the elimination of debris. The obtained lysate was quantized by BioSpectometer (Eppendorf, Germany) and a concentration of 50 µg was used for sodium dodecyl-sulfate polyacrylamide gel electrophoresis (SDS-PAGE) followed by Western blot analysis (Bio-Rad V3 Western Workflow™, Milan, Italy). The transfer procedure was realized with semidry technique on PVDF membranes (Immobilon‐P transfer membrane; Millipore). The saturation was performed incubating the membranes with 1× PBS, 5% milk and 0.1% Tween for 2 h at RT. The primary antibodies anti-Ki-67 (1:500 mouse; BioGenex, Fremont, CA, USA), anti-COL1A1 (1:1000 rabbit; Invitrogen, MA, USA), anti-RUNX-2 (1:500 mouse; Santa Cruz Biotechnology,CA, USA) and anti-OPN (1:500 mouse; Santa Cruz Biotechnology) were inserted overnight at 4 °C. Respectively, the peroxidase-conjugated anti-mouse secondary antibody and anti-rabbit secondary antibody (1:5000; Bethyl Laboratories, Montgomery, AL, USA) were finally incubated at RT for 1 h. Eventually, the membranes were read using the Alliance 2.7 system (Uvitec Ltd, Cambridge, UK), which allowed identifying and quantifying the bands obtained. The data achieved were normalized with the protein expression of β-actin (1:750 mouse, Santa Cruz Biotechnology).

### Statistical analysis

All results were expressed as mean ± standard deviation. For repeated measures, ANOVA was performed to compare means between groups.

The ‘fold change’ of gene expression levels was calculated with the 2^−ΔΔCt^ method. The hypothesis that the fold change between exposed and not exposed was equal to 1 was tested with the Student’s *t*-test for unpaired data.

## Results

### Cellular characterization

**Fig. 3:**
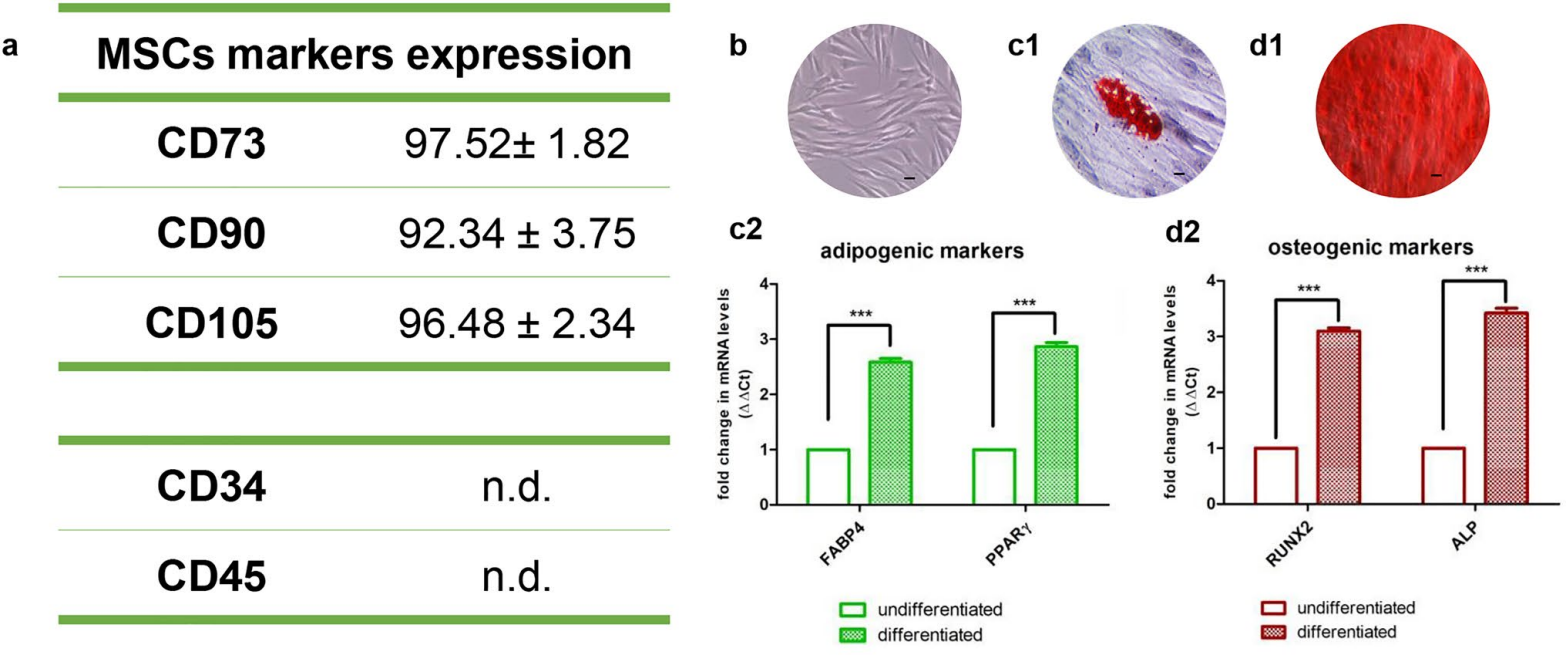
Human periodontal ligament stem cells. **a** hPDLSCs were positive for CD73, CD90 and CD105 but negative for CD34 and CD45. **b** hPDLSCs observed under light microscopy showed fibroblast phenotype features. (c1, c2) hPDLSCs showed potential for differentiation toward adipogenic (Oil Red O staining) lineage. (**d1**,** d2**) hPDLSCs showed potential for differentiation toward osteogenic (Alizarin Red) lineage. Scale bar: 20 µm. ***p < 0.001

As shown in Fig. 2, the flow cytometry of hPDLSCs revealed the positive expression of CD73, CD90 and CD105 and the negative expression of CD34 and CD45. The pluripotency of hPDLSCs was confirmed by the capacity to differentiate into adipogenic and osteogenic lineages in vitro as observed under light microscopy (Fig. 3c1, d1) and confirmed by gene expression (Fig. 3c2, d2). Plastic adherent cells showed a fibroblast-like morphological features (Fig. 3b).

### Effects of ELF-EMF on cell morphology, cell proliferation and de novo calcium deposition

The effect of ELF-EMF exposure on morphological changes of hPDLSCs cells after experimental treatments were evaluated by optical microscopy. When cells were cultured without osteogenic culture medium (without OM) and exposed to sham condition, hPDLSCs grew free from calcium deposition.

In cells cultured with OM, as expected, de novo calcium deposition was detected, indicating osteoblastic differentiation. Differently, for cells exposed to ELF-EMF, we observed an osteoblastic morphology, both in the presence or not, of OM, suggesting that the effect can be field-related.

**Fig. 4:**
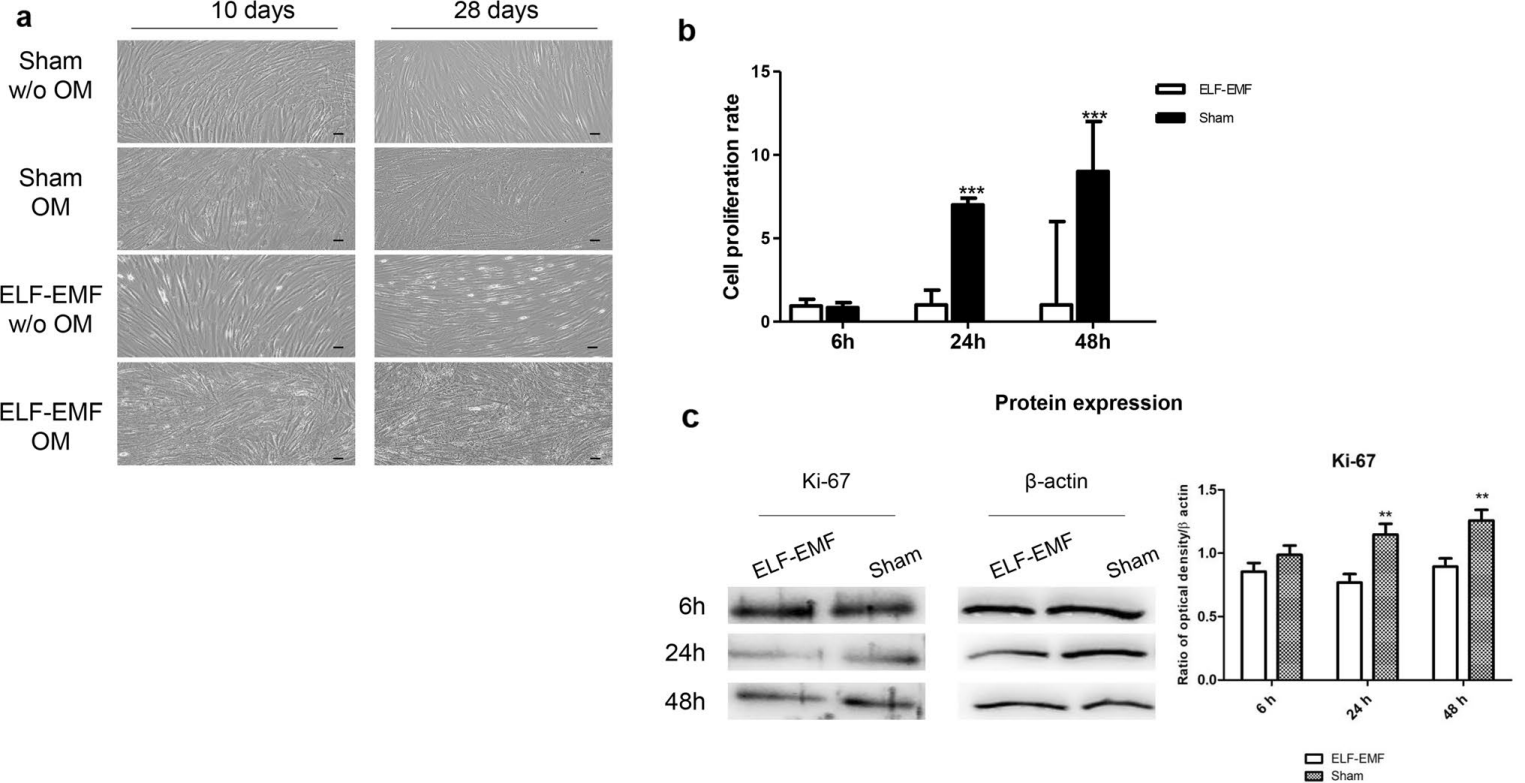
**a** Microphotographs of hPDLSCs cultured in the presence of experimental conditions for 10 and 28 days. Images were obtained using an inverted microscope (Leica DMi1, Wetzlar, Germany). Scale bar: 20 µm. **b** MTT assay was performed on hPDLSCs in presence of no-OM, and exposed to ELF-EMF or sham system, for 6, 24 and 48 h. **c** Western blot analysis of Ki-67 expression. Data are expressed as mean ± SD of two experiments performed in triplicate. ***p < 0.001

Moreover, an interesting result was that ELF-EMF exposure improved the osteogenic differentiation; indeed, after 10 days of culture, morphological changes were observed in bot the presence and absence of OM (Fig. 4a).

To evaluate the impact of ELF-EMF exposure on cell viability, MTT assay was performed. Our results showed that in hPDLSCs cultured for 24 and 48 h, cell proliferation rate was significantly higher than the proliferation observed in sham exposed cells (Fig. 4b).

**Fig. 5:**
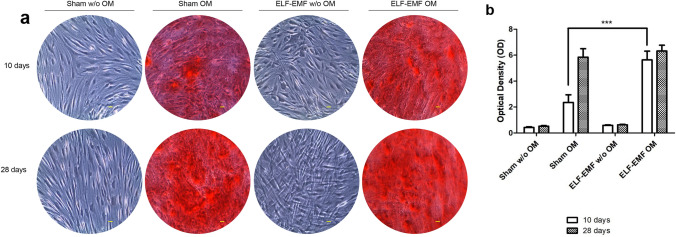
hPDLSCs maintained in different culture conditions, sham without OM, sham OM, ELF-EMF without OM and ELF-EMF OM, were stained using Alizarin Red S solution after 10 and 2 days of culture. **a** Images obtained by light microscopy. **b** Bar graph shows the Alizarin Red S quantization. ***p < 0.001. Images are representative of three independent experiments. Scale bar: 20 μm

To better evaluate the effect of ELF-EMF exposition on cell culture, the expression of Ki-67 was demonstrated by Western blot analysis. At 24 and 48 h of culture, the Ki-67 expression was high in cells exposed to sham conditions compared to ELF-EMF cells (ELF.EMF vs sham at 24 h and 48 h *p* < 0.01) (Fig. 4c). The effect on the osteogenic process was evaluated by the morphological analyses obtained after Alizarin red S (ARS) staining and its quantification. Cells maintained under ELF-EMF exposure in the osteogenic medium showed a strong positivity for ARS staining after 10 days of exposure at the same level as obtained after 28 days of culture. Cells maintained under sham effect showed a low positivity at 10 days compared to the ELF-EMF. No differences were evaluated after 28 days of exposure to ELF-EMF and/or sham observed under light microscopy (Fig. 5a). These qualitative data were confirmed by alizarin red quantification analysis (Fig. 5b).

### Effects of ELF-EMF on osteogenic marker gene expression

To analyze the effects of ELF-EMF exposure on the gene expression pattern of hPDLSC cell growth with or without osteogenic medium for 10 and 28 days, qRT-PCR was performed.

**Fig. 6:**
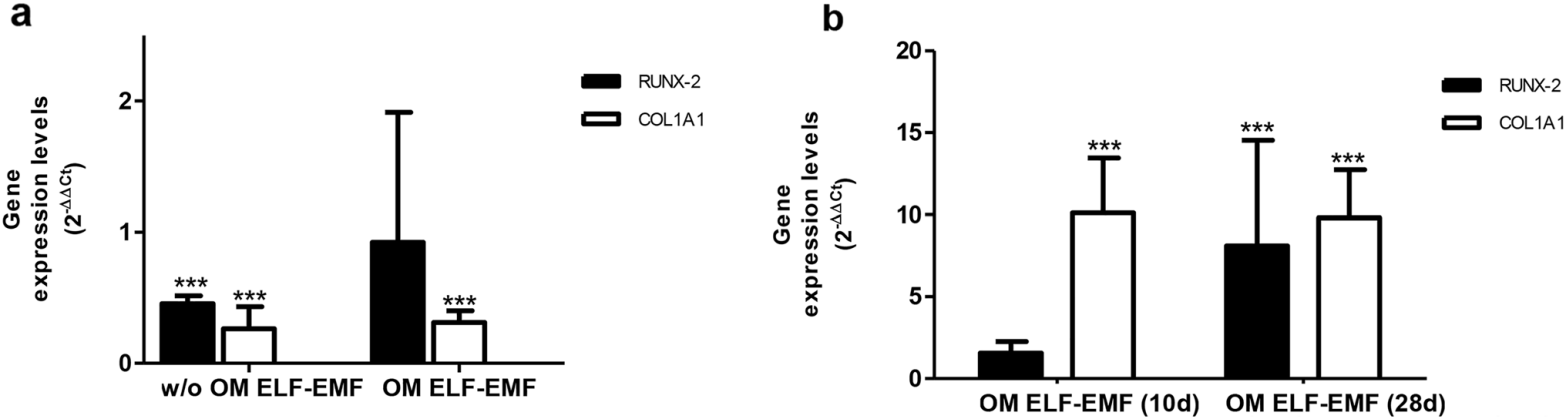
**a** The mRNA expression of RUNX-2 and COL1A1 in hPDLSCs exposed to ELF-EMF for 28 days compared to cells exposed to ELF-EMF for 10 days, both with and without OM. ***p < 0.001 28 days vs 10 days assumed as 1. **b** The mRNA expression of RUNX-2 and COL1A1 in hPDLSCs exposed to ELF-EMF for 28 and 10 days compared to sham-exposed cells. Expression levels (2^−∆∆Ct^) are reported as mean ± 95% CI of two independent experiments. ***p < 0.001 ELF-EMF vs sham assumed as 1

A significant downregulation of RUNX-2 and COL1A1 osteogenic markers was observed in hPDLSCs exposed to ELF-EMF for 28 days compared to 10 days of exposure, both with and without OM, suggesting the ability of ELF-EMF to improve new bone formation early (Fig. 6a).

To confirm that the variation of osteogenic marker expression was related to ELF-EMF exposure, we analyzed the gene expression levels in hPDLSCs exposed to ELF-EMF in presence of OM, for 10 and 28 days, in relation to the sham-exposed cells. As reported in Fig. 6b, after 10 days of ELF-EMF exposure, in OM-cultured hPDLSCs, a significant upregulation of COL1A1 was observed compared to sham-exposed cells. Moreover, a significant upregulation of RUNX-2 and COL1A1 (7.1- and 8.8-fold increase, respectively) was also observed in hPDLSCs ELF-EMF exposed for 28 days compared to sham exposure condition. These data support the potential empowering action of ELF-EMF exposure to favor osteoblastic differentiation.

### Effects of ELF-EMF on osteogenic markers protein expression

**Fig. 7:**
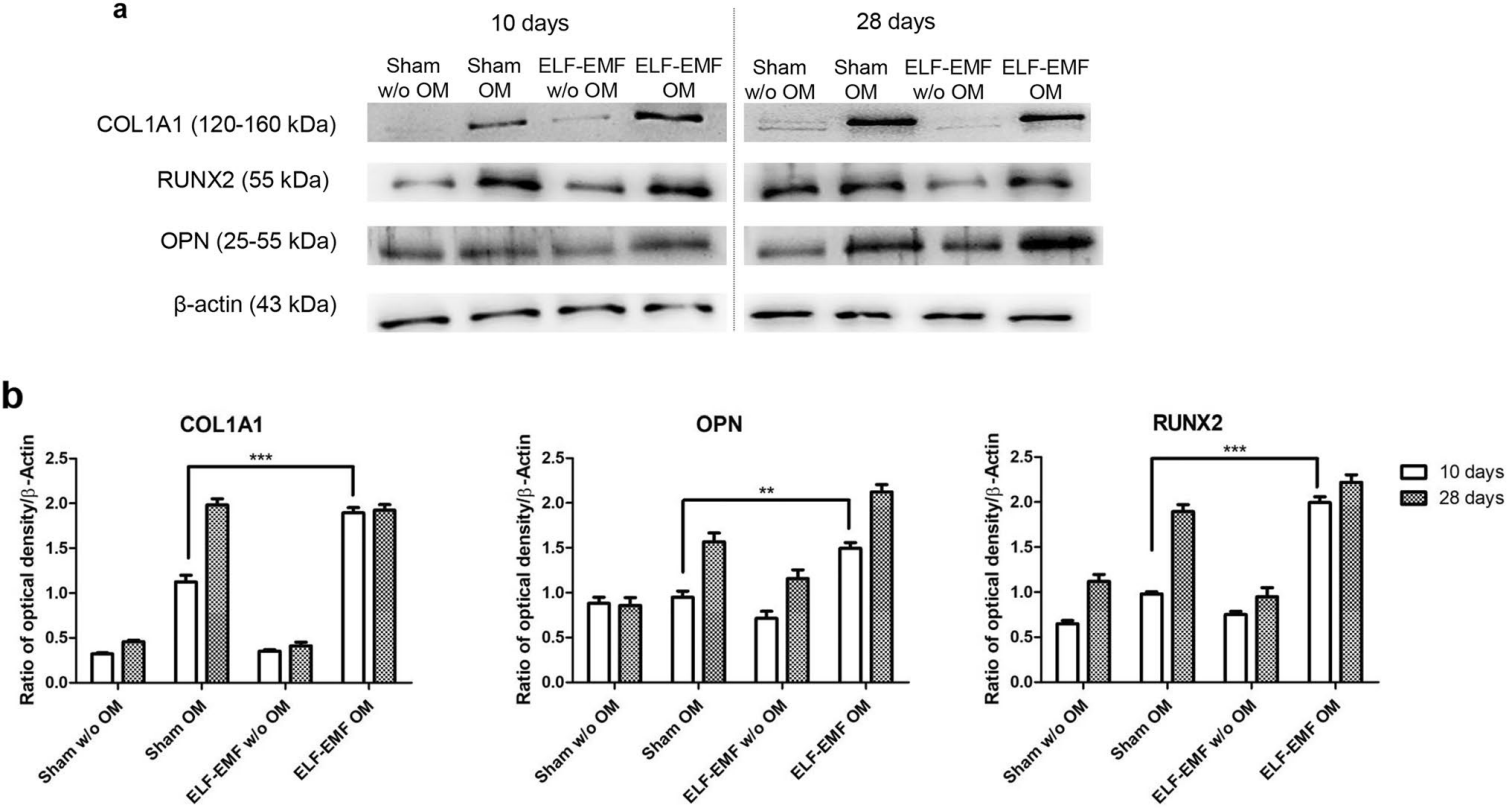
**a** Specific bands of protein levels of COL1A1 and RUNX-2 in all samples evaluated after 10 and 28 days of culture. Beta actin was used as housekeeping protein. **b** Relative ratio of COL1A1 and RUNX-2 normalized with β-actin. Images are representative of three independent experiments. ***p < 0.001

**Fig. 8:**
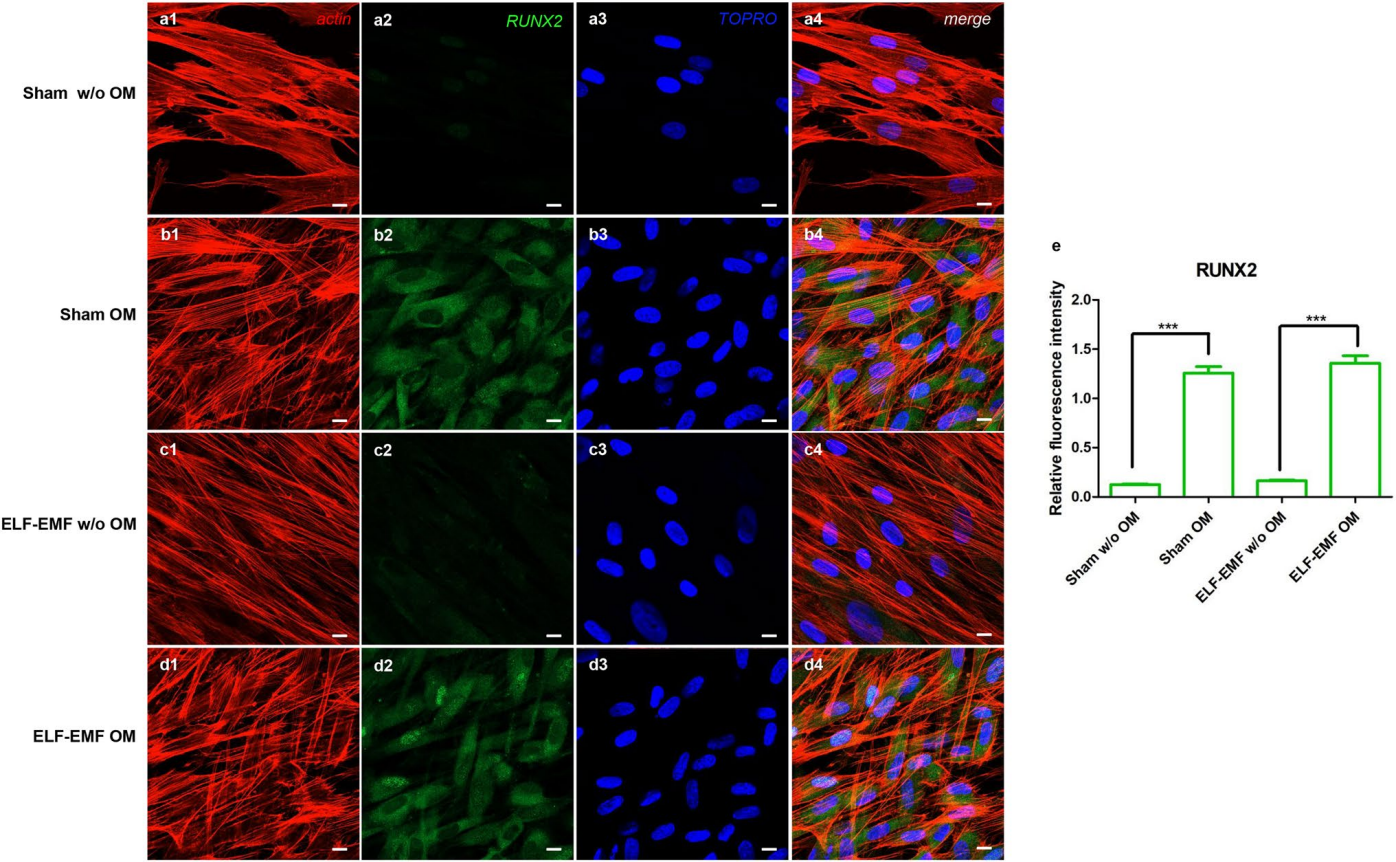
RUNX-2 expression in hPDLSCs maintained in different culture conditions after 10 days of culture: (**a1**–**a4**) sham w/o OM, (**b1**–**b4**) sham OM, (**c1**–**c4**) ELF-EMF w/o OM and (**d1**–**d4**) ELF-EMF OM—was evaluated at confocal laser scanning microscopy analysis. Red fluorescence: cytoskeleton actin. Green fluorescence: RUNX-2. Blue fluorescence: cell nuclei. Images are representative of three independent experiments. **e** The relative fluorescent intensity of RUNX-2 was analyzed using NIS-Elements AR imaging software. Data are presented as the mean of 30 measurements ± standard deviation. **p < 0.01. Scale bar: 10 μm

To evaluate the effects of ELF-EMF exposure on protein expression, immunofluorescence investigation and Western blot analysis were performed. The bar graph in Fig. 7b shows the densitometric analysis of the specific bands of osteogenic-related markers, COL1A1, RUNX-2 and OPN (COL1A1 expression of sham OM vs ELF-EMF OM, ***p < 0.001; RUNX-2 expression of sham OM vs ELF-EMF OM, ***p < 0.001; OPN expression of sham OM vs ELF-EMF OM, **p < 0.01). A significant expression of osteogenic markers was detected in OM-cultured hPDLSCs exposed to ELF-EMF with respect to sham-exposed OM-cultured hPDLSCs after 10 days of culture. No statistical differences were evidenced between ELF-EMF and sham exposed OM-cultured hPDLSCs after 28 days of culture. This data could suggest an important role of ELF-EMF exposure during the early stage of differentiation of cells maintained under osteogenic conditions (Fig. 7). The confocal laser scanning microscopy images showed the expression of RUNX-2 in sham and ELF-EMF OM-cultured hPDLSCs after 10 days of culture, suggesting that ELF-EMF exposure facilitates the differentiation process (Fig. 8b2, d2). Graph bars of quantification of RUNX-2 expression showed high expression in cells exposed to ELF-EMF OM after 10 days of culture, demonstrating the importance of the electromagnetic field in stimulation of the bone regeneration process (Fig. 8e).

## Discussion

Osteogenic differentiation is a progressive physiological process in which specific genes and proteins are differently expressed in a temporal and coordinating manner, according to morphological, cytochemical and biochemical changes.

Previous studies on MSCs differentiation have underlined the ability of EMF to induce osteogenic differentiation, triggering the induction of morphological and transcriptional and post-transcriptional changes (Maziarz et al. 2016). The ability of ELF-EMF to drive phenotypical differentiation was widely demonstrated in different stem cells lines, such as human bone marrow mesenchymal stem cells (BMMSC) (Sun et al. 2009), human osteosarcoma cells (Mansourian and Shanei 2021) and human adipose derived mesenchymal stem cells (Poh et al. 2018) while only few studies have evaluated the effects of ELF-EMF on hPDLSCs (Wang et al. 2017b; Li et al. 2021). In this study, we investigated not only the effects of ELF-EMF on the osteogenic differentiation of hPDLSCs, but also the cellular response over time.

hPDLSCs derived from human periodontal ligament tissue can be isolated with minimally invasive techniques during routine dental procedures. Emerging evidence shows that hPDLSCs have excellent anti-inflammatory and immunomodulatory effects and can be considered an excellent in vitro model to study the mechanism of the bone regeneration process (Pizzicannella et al. 2021a).

To characterize this type of cells, the International Society for Cellular Therapy set the minimal criteria. In particular, MSCs are plastic adherent with a fibroblastic morphology, show potential to differentiate into osteogenic, chondrogenic and adipogenic lineages, express cell surface molecules such as CD73, CD90 and CD105, and lack expression of hematopoietic and endothelial antigens (Dominici et al. 2006).

Human PDLSCs were intermittently exposed to ELF-EMF for 6 h/day, for 10 and 28 days, to compare the effects on cell growth and differentiation. Our results pointed out the ability of ELF-EMF to induce an early morphological differentiation of hPDLSCs into osteogenic cells, already evident after 10 days.

It is well know that osteogenic differentiation is regulated by multiple gene signaling pathways that lead to the upregulation of the transcriptional activity of RUNX-2. Furthermore, RUNX-2 can upregulate BMP2, an early marker of osteogenesis, and enhance the expression of late osteogenic differentiation genes (Ren et al. 2022), which is considered an essential factor for the proliferation of osteoprogenitor cells during the differentiation of osteoblasts (Helaehil et al. 2021). Several papers have speculated that osteoblasts and fibroblasts differentiated from mesenchymal stem cells are the major source of COL1 in various organs. Type I collagen (COL1) is the most abundant protein in the bone tissue and is involved in the development, formation, homeostasis and remodeling of bone tissue (Chen et al. 2021). Osteopontin (OPN) is considered a marker of the terminal stage of osteoblast differentiation and is a prominent bone matrix protein (Niknam et al. 2022). Our results showed that osteogenic genes and protein expression were significantly increased in RUNX-2 and COL1A1 levels after 10 days of exposure, and the early synthesis of collagen in hPDLSCs and following insoluble collagen deposition can be regulated by ELF-EMF, facilitating osteogenic differentiation by increasing both collagen type 1 gene expression and protein secretion (Li et al. 2021).

Stimulation with EMF increased the intracellular calcium concentration by regulating calcium channels, which are required for the MSC differentiation (Yan et al. 2021). Recently, Parate et al. showed that MSC-derived conditioned medium post-EMF exposure could affect the paracrine function of MSCs (Parate et al. 2020). EMF can regulate Wnt1/β-catenin signaling pathway involved in osteogenesis and bone metabolism (Catalano et al. 2018; Zhang et al. 2020).

Based on our results in this study, we further verified that ELF-EMF can facilitate osteogenic differentiation and contribute to a decreased induction time process by promoting the expression of RUNX-2 and COL1A1.

## Conclusion

ELF-EMF exposure may represent an innovative method for osteogenic differentiation and stabilization of hPDLSCs. The ability to increase the multi-potency and immune modulatory properties of hPDLSCs and the greater ability to differentiate in the presence of ELF-EMF make these cells the “gold” candidates for regenerative medicine, especially in the field of bone engineering. Collectively, our data highlight the signaling modulation of ELF-EMF on hPDLSCs, encouraging further investigations on the use of ELF-EMF for bone repair therapy approaches.

## Data Availability

Data are available from the corresponding author upon request.
